# Acetonitrile-Based Antisolvent Strategy for Performance Enhancement of CsPbBr_3_ Perovskite Solar Cells

**DOI:** 10.3390/mi17091009

**Published:** 2026-08-26

**Authors:** Ying Chen

**Affiliations:** School of Science, Hubei University of Technology, Wuhan 430068, China; chenying@hbut.edu.cn

**Keywords:** CsPbBr_3_, perovskite solar cells, acetonitrile, antisolvent

## Abstract

Inorganic CsPbBr_3_ perovskite has garnered significant research interest owing to its exceptional stability under harsh humid conditions; however, the power conversion efficiency (PCE) of CsPbBr_3_-based perovskite solar cells (PSCs) remains lower than that of their hybrid counterparts. Antisolvent-assisted crystallization is a robust strategy for fabricating high-quality perovskite films for high-performance PSCs. In this study, high-quality CsPbBr_3_ perovskite films were prepared by incorporating an optimized concentration of acetonitrile (ACN) into the PbBr_2_ precursor solution under ambient air conditions. The resulting CsPbBr_3_ perovskite layer exhibited enlarged grain size, reduced surface defect density, and enhanced charge-carrier transport properties. Consequently, carbon-based, hole transport layer-free CsPbBr_3_ PSCs achieved a PCE increase from 2.63% to 6.69%. This research presents an effective antisolvent engineering strategy for high-quality film fabrication and offers valuable insights for advancing the commercial viability of PSCs.

## 1. Introduction

With the progressive depletion of fossil fuel reserves and the exacerbation of environmental pollution, the exploitation of renewable energy has become increasingly imperative. Among various alternatives, solar energy stands out as a virtually inexhaustible and clean resource with considerable promise. Perovskite solar cells (PSCs) have garnered substantial research interest owing to their exceptional light absorption coefficient, prolonged carrier lifetime, low cost, and facile processing protocols [1,2,3,4,5]. Various photovoltaic materials, including silicon, chalcogenides, organics, and binary oxide ceramics, are being explored for solar-cell applications [6]. In particular, inorganic CsPbBr_3_ perovskite exhibits remarkable tolerance to moisture, oxygen, and elevated temperatures, rendering it highly promising for applications in tandem and semi-transparent photovoltaic devices [7,8,9,10,11,12]. Nevertheless, the power conversion efficiency (PCE) of CsPbBr_3_-based PSCs remains severely constrained by suboptimal film quality. Consequently, the development of effective strategies to modulate the crystallization behavior of CsPbBr_3_ thin films is of critical importance.

Multiple effective approaches have been reported to improve the performance of CsPbBr_3_ perovskite solar cells, including additive engineering, interface passivation [13], and plasmonic nanoparticle incorporation. Plasmonic components embedded in perovskite precursors can enhance light harvesting and modulate exciton dissociation [14]. Solvent engineering is another facile route to tune nucleation and grain growth [15]. This approach typically involves depositing a precursor mixture, which comprises halide salts dissolved in organic solvents, onto a substrate via spin coating or blade coating. Several studies [16,17,18,19] used dimethyl sulfoxide (DMSO) and dimethylformamide (DMF) as co-solvents to fabricate CsPbI_2_Br films, thereby enhancing the solubility of CsPbI_2_Br precursors. Meng et al. [20] employed a binary solvent system comprising ethylene glycol monomethyl ether (EGME) and isopropanol (IPA) to modulate the Ostwald ripening process during film formation. This controlled crystallization strategy yielded high-quality CsPbBr_3_ films with enhanced crystallinity and favorable morphological properties. He et al. [21] demonstrated that isopropanol and thiourea effectively enhanced the crystallinity of CsPbIBr_2_ films, yielding a PCE of 6.79% for the carbon-based CsPbIBr_2_ PSCs, an approximate 30% improvement over untreated devices. Wang et al. [22] replaced toxic methanol with environmentally benign water and ethylene glycol monomethyl ether to fabricate high-quality CsPbBr_3_ films. Han et al. [23] optimized CsPbI_3_ film performance using ethyl acetate. Yang et al. [24] employed a chlorobenzene/isopropanol (CB/IPA) mixed antisolvent system to improve CsPbIBr_2_ film quality, achieving a champion PCE of 8.48% in FTO/TiO_2_/CsPbIBr_2_/ZnPc/carbon-structured devices. Zhou et al. [25] utilized a methylamine/acetonitrile solution to heal grain boundary defects in MAPbI_3_ perovskite.

In contrast to previously reported high-dose ACN strategies or post-treatment approaches, this work incorporates low doses of ACN (2–8 vol%) directly into the PbBr_2_/DMF precursor solution to facilitate the crystallization of CsPbBr_3_ films. The effect of ACN addition on film morphology and defect density was systematically examined. At the optimal concentration of 5 vol% ACN, intragranular defects and grain boundary imperfections were substantially reduced. Consequently, the resulting carbon-based, hole-transport-layer-free CsPbBr_3_ perovskite solar cell achieved a champion power conversion efficiency of 6.69%, outperforming both the pristine device (2.63%) and the conventionally ACN-post-treated device (3.69%).

## 2. Experimental

### 2.1. Materials

Cesium bromide (CsBr; 99.999%) and lead bromide (PbBr_2_; 99.999%) were purchased from Xi’an Polymer Light Technology (Xi’an, China). Isopropyl titanate (C_16_H_28_O_6_Ti; 99%) and carbon paste (<30 Ω) were obtained from Shanghai Mactowell Chemical New Material Technology Co., Ltd. (Shanghai, China). Isopropyl titanate (AR), N, N’-dimethylformamide (DMF; 99%), acetonitrile (ACN; AR), acetone (AR), isopropanol (AR), ethyl alcohol (AR), n-butanol (99.5%), methyl alcohol (99%), and anhydrous methanol (AR) were supplied from Sinopharm Group Chemical Co., Ltd. (Shanghai, China). Fluorine-doped tin oxide (FTO) conducting glass (FTO; 15 Ω, 2 cm × 2 cm) and the detergent were purchased from Liaoning Preferred New Energy Technology Co., Ltd. (Yingkou, China).

### 2.2. Device Preparation

All preparation procedures were conducted under ambient atmospheric conditions without humidity control. FTO conductive glass substrates were sequentially ultrasonicated in detergent, deionized water, isopropanol, and ethanol for 20 min each. The cleaned substrates were then dried under a nitrogen stream and subjected to UV-ozone treatment for 30 min to improve surface hydrophilicity. An isopropyl titanate/ethanol solution (0.15 M) was subsequently spin-coated onto the treated FTO substrates at 4500 rpm for 30 s, followed by annealing at 450 °C for 30 min to yield a compact TiO_2_ layer.

**Figure 1:**
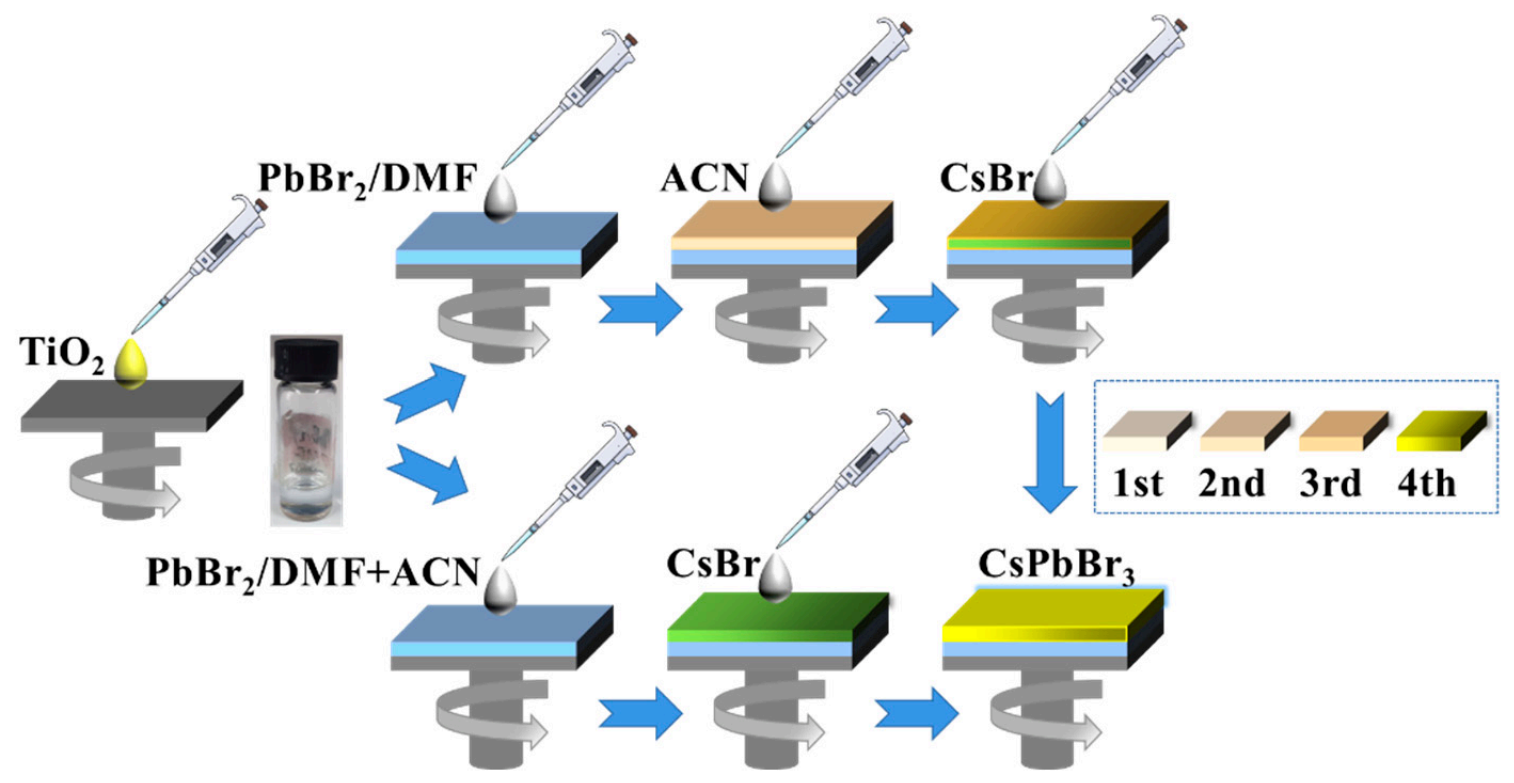
Fabrication process flowchart for a CsPbBr_3_ perovskite solar cell.

For perovskite film fabrication (Figure 1), ACN antisolvent with varying concentrations (0 vol%, 2 vol%, 5 vol%, and 8 vol%) was directly added to the PbBr_2_/DMF solution (5 M), followed by spin-coating on the substrate at 2000 rpm for 30 s and annealing at 90 °C for 30 min. A CsBr/methanol solution (0.07 M) was then spin-coated on the as-treated substrate at 2000 rpm for 30 s, followed by annealing at 250 °C for 5 min. This two-step deposition-annealing cycle was repeated 4–5 times to afford a uniform CsPbBr_3_ film. In the conventional ACN anti-solvent post-treatment protocol, the substrate was sequentially coated with the PbBr_2_/DMF precursor solution, ACN anti-solvent, and CsBr precursor solution. Finally, a conductive carbon electrode was blade-coated onto the as-prepared CsPbBr_3_ film (active area: 0.1 cm^2^) and annealed at 100 °C for 30 min to complete device fabrication.

### 2.3. Device Characterization

X-ray diffraction (XRD-7000, Shimadzu, Kyoto, Japan) was conducted under Cu-Kα radiation (λ = 1.5418 Å) in a scanning range of 20–50° to analyze the crystal structures of the as-developed CsPbBr_3_ films. Field-emission scanning electron microscopy (FESEM; SU8010, Hitachi, Tokyo, Japan) was performed at an acceleration voltage of 15 kV to observe the microstructural morphology of the CsPbBr_3_ films. The elemental compositions of the films were identified by X-ray photoelectron spectroscopy (XPS) under Al-Kα excitation (1486.6 eV). UV-visible spectrophotometry (UV-vis; U-3900, Hitachi, Japan) was carried out to test the light absorption capacity of the films. Steady photoluminescence (PL) and time-resolved photoluminescence (TRPL) decay measurements were conducted using a steady-state fluorescence spectrometer (FLS 980, Edinburgh Instruments, Livingston, UK) and a time-of-flight fluorescence spectrometer (FLS1000, Edinburgh Instruments, UK), respectively. A solar simulator (LZB54AR008-11, Atlas MTT GmbH, Linsengericht, Germany) and a digital source meter (Keithley 2420, Tektronix, Beaverton, OR, USA) were used to test the *J*-*V* curves of the solar cells, and light irradiance of AM 1.5 G (100 mW·cm^−2^) was used in the atmospheric environment. The films were calibrated with a standard silicon cell before testing, and electrochemical impedance spectroscopy (EIS) data were measured using an electrochemical workstation (VearsaSTAT3, Princeton, NJ, USA).

## 3. Results and Discussion

**Figure 2:**
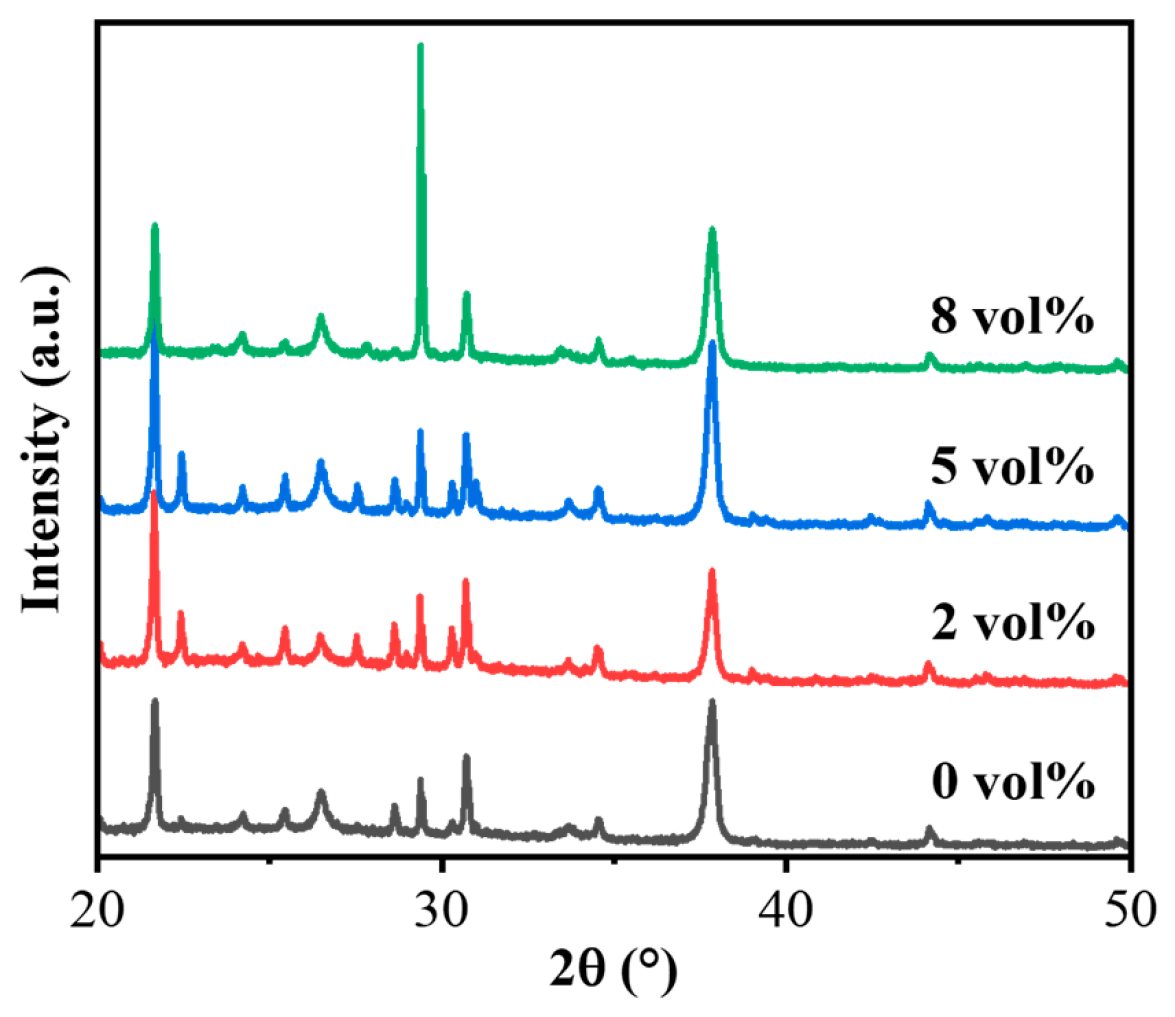
XRD patterns of the CsPbBr_3_ perovskite films fabricated with varying ACN volume fractions.

Figure 2 displays the XRD patterns of the CsPbBr_3_ films fabricated with varying ACN volume fractions. The characteristic diffraction peaks of CsPbBr_3_ were observed at 21.50°, 30.78°, and 37.78°, consistent with the standard card (PDF#18-0364 [26]). The diffraction peak intensity exhibits a pronounced dependence on the ACN content. Specifically, the intensity increases progressively as the ACN volume fraction rises from 0 to 5%. However, upon reaching 8%, the peak intensity diminishes gradually, accompanied by the emergence of a new diffraction peak corresponding to the CsPb_2_Br_5_ impurity phase. The phenomenon is attributed to excessive ACN addition, which induces over-extraction of PbBr_2_ from the precursor solution, thereby altering the PbBr_2_ concentration and promoting the formation of the secondary phase via reaction with CsBr. XRD patterns were quantitatively analyzed using MDI-Jade 6. After background subtraction and Kα_2_ stripping, instrumental broadening was corrected with a silicon-derived IPC file. High-signal representative peaks were fitted by the pseudo-Voigt function to obtain FWHM and fitting uncertainties. Crystallite domain sizes were calculated by the Scherrer equation (K = 0.89). Note that Scherrer values represent coherent scattering domains instead of physical particle sizes. Preferred-orientation evaluation used all observable diffraction intensities against PDF reference data. No obvious orientation difference was found after ACN treatment. The narrower FWHM and stronger diffraction intensities therefore arise from larger crystallite domains and better crystallinity. Quantitative results are given in Table 1.

**Figure 3:**
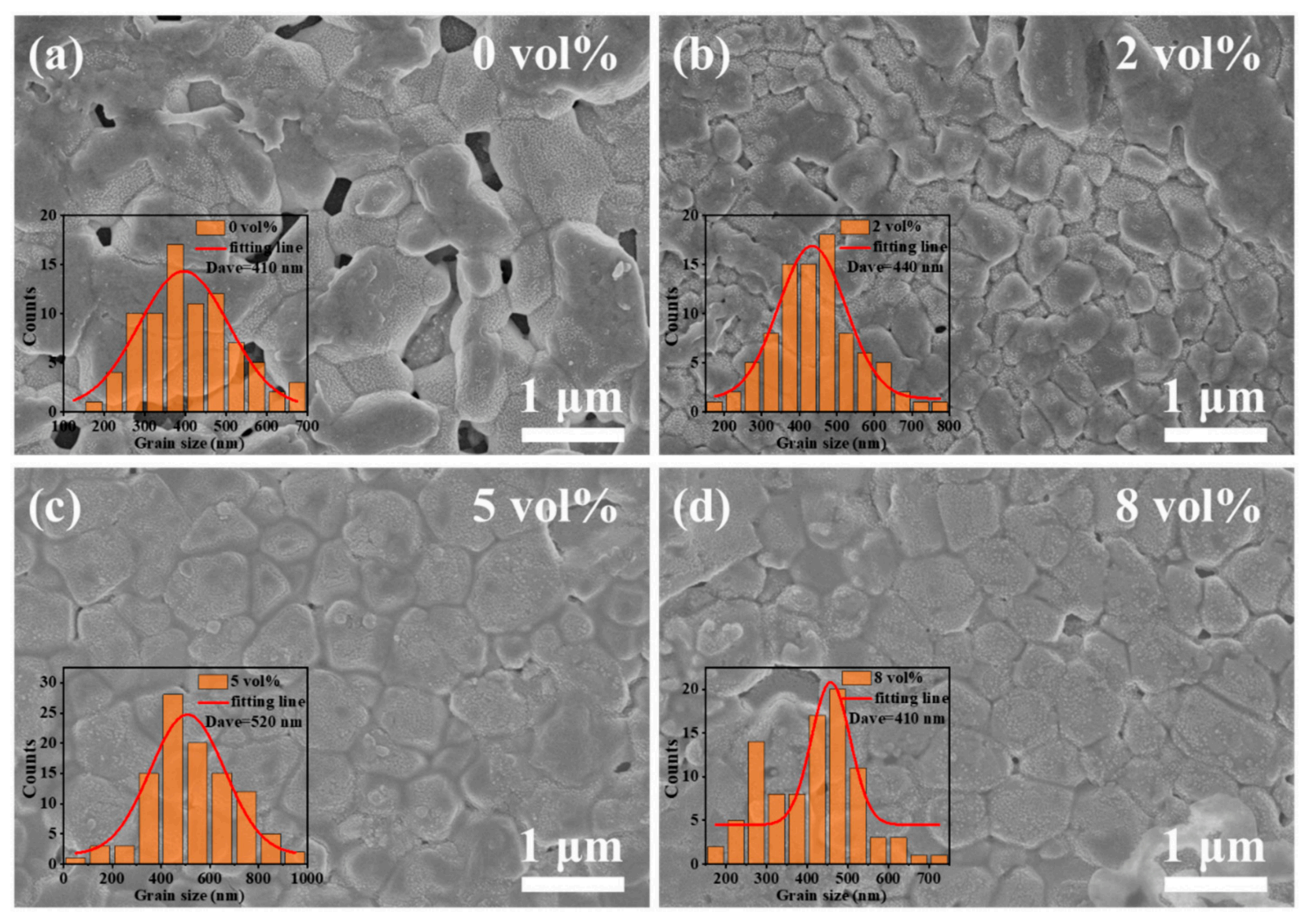
SEM images of the CsPbBr_3_ films fabricated with varying ACN volume fractions: (**a**) 0 vol%, (**b**) 2 vol%, (**c**) 5 vol%, and (**d**) 8 vol%. The insets display the corresponding grain size distribution histograms.

Figure 3 presents the SEM morphologies of the CsPbBr_3_ perovskite films fabricated with varying ACN volume fractions. Statistical grain size analysis, performed using Nano Measurer software, is summarized in Table 2. The perovskite film without ACN (0 vol%) exhibited abundant pinholes and defects with an average grain size of 410 nm. Upon introducing 2 vol% ACN, the density of pinholes and defects decreased markedly, resulting in a compact film morphology. Further increasing the ACN content to 5 vol% yielded a denser film with uniformly distributed, well-defined grains, where the average grain size reached 520 nm. Concurrently, grain boundaries were reduced and pinholes/defects were effectively eliminated. However, at 8 vol% ACN, pinholes re-emerged within tightly packed grain interstices and the average grain size regressed to 410 nm. This deterioration is attributed to excessive solvent extraction by ACN, which induced rapid solute precipitation. The precipitated species accumulated extensively at grain boundaries, suppressing CsPbBr_3_ grain growth during annealing. Consequently, the reduced grain size and increased grain boundary density facilitated the formation of pinholes and defects. Notably, this interpretation is a hypothesis supported by indirect XRD and device-performance evidence. Direct compositional measurements of precursor/intermediate films will be explored in future studies.

**Figure 4:**
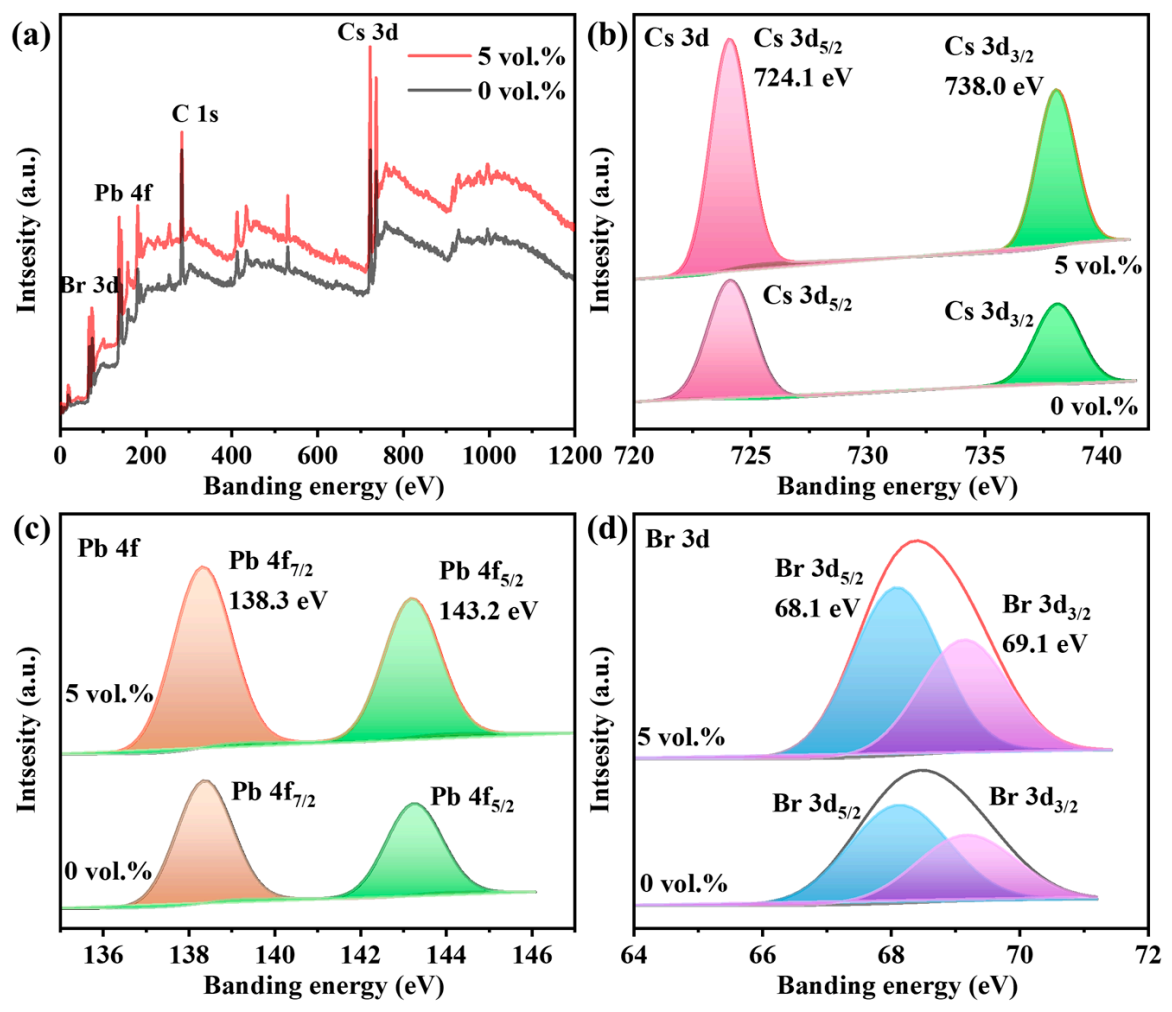
XPS spectra of CsPbBr_3_: (**a**) full CsPbBr_3_ spectrum, (**b**) Cs 3d spectrum, (**c**) Pb 4f spectrum, and (**d**) Br 3d spectrum.

To elucidate the influence of ACN incorporation on the physicochemical properties of the perovskite films, XPS spectra of the CsPbBr_3_ films prepared with 0 vol% and 5 vol% of ACN are comparatively analyzed (Figure 4). As shown in Figure 4a, regardless of ACN addition, only the characteristic peaks corresponding to Cs 3d, Pb 4f, Br 3d, and C1s were detected, indicating that ACN did not alter the elemental composition of the films. Most ACN is eliminated during the post-deposition thermal annealing process. Detecting trace residual ACN-derived species would require more sensitive analytical techniques. Figure 4b–d) exhibit the high-resolution XPS spectra of the Cs 3d, Pb 4f, and Br 3d orbitals, respectively. With charge referencing by adventitious C 1s (284.8 eV), no obvious binding-energy shifts are observed for Cs 3d, Pb 4f and Br 3d. The main spectral variation is the reduced FWHM, revealing a more homogeneous local chemical environment on the film surface. This demonstrates that ACN treatment mitigates lattice distortion and surface disorder, consistent with enhanced crystallinity. Fitting details are summarized in Table 3.

**Figure 5:**
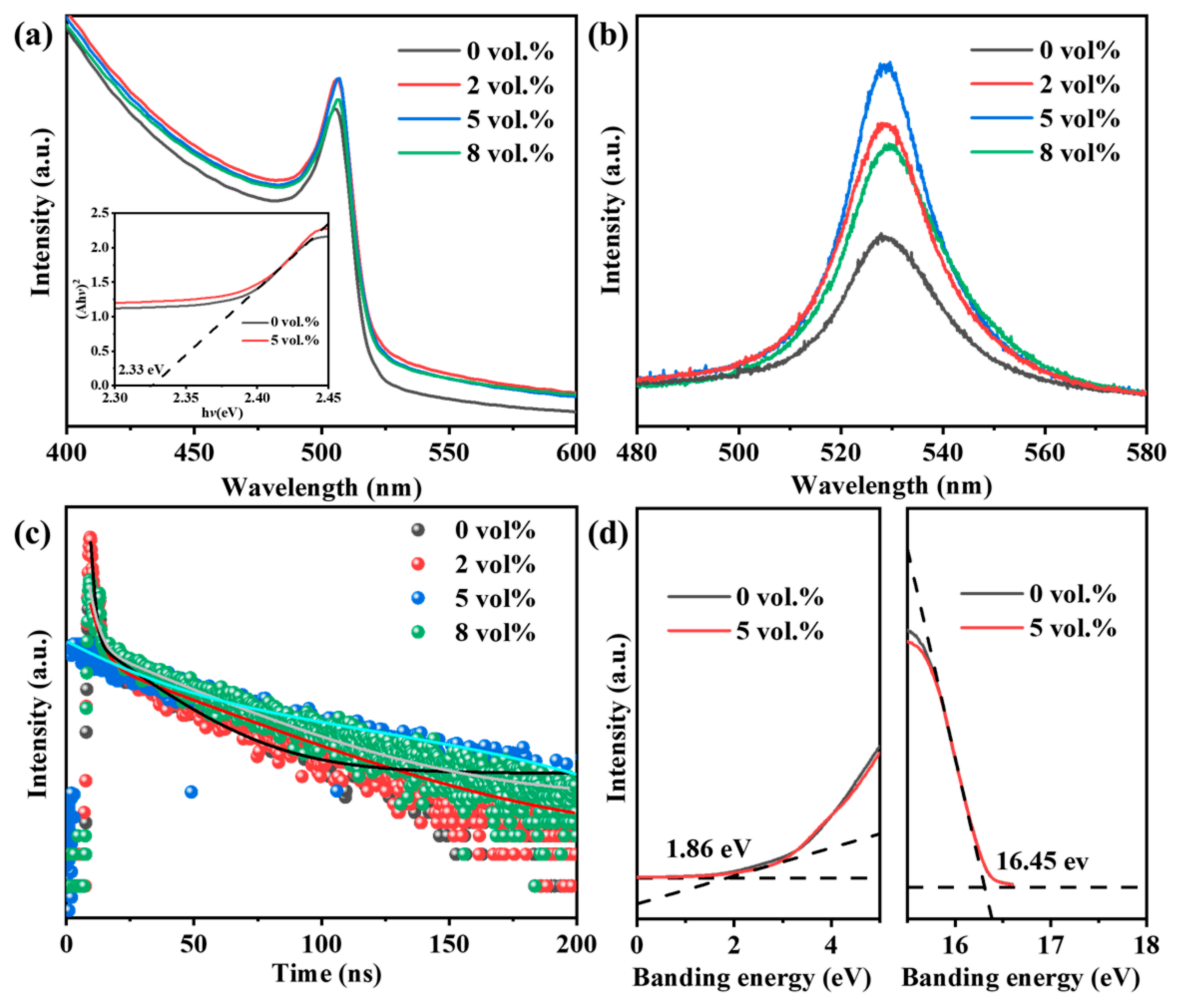
(**a**) UV-vis absorption spectra with corresponding Tauc plots (insets), (**b**) PL spectra, (**c**) TRPL curves, and (**d**) onset energy (*E_onset_*) region and cutoff energy (*E_cutoff_*) region of various CsPbBr_3_ films.

Figure 5a presents the UV-vis absorption spectra of the CsPbBr_3_ films fabricated with varying ACN volume fractions, with the inset showing the corresponding Tauc plots. All samples exhibit a nearly identical absorption onset at approximately 523 nm, corresponding to a bandgap of 2.33 eV [27]. As shown in the steady-state PL spectra in Figure 5b, the band-to-band radiative recombination peaks at ~528 nm [28]. Notably, the incorporation of 5 vol% ACN yields the highest PL intensity among all compositions. The time-resolved photoluminescence (TRPL) spectra of the as-prepared films are depicted in Figure 5c and were fitted using a double-exponential function:

(1) $$I(t)=A_{1}e^{\frac{-t}{\tau_{1}}}+A_{2}e^{\frac{-t}{\tau_{2}}}$$

where *A*_1_ and *A*_2_ denote the pre-exponential factors, and *τ*_1_ and *τ*_2_ represent the time constants. Double-exponential fitting yielded *τ*_1_ = 3.87 ns and *τ*_2_ = 48.60 ns for the perovskite film without ACN (0 vol%), *τ*_1_ = 1.09 ns and *τ*_2_ = 23.52 ns for the 2 vol% ACN film, *τ*_1_ = 5.83 ns and *τ*_2_ = 59.65 ns for the 5 vol% ACN film, and *τ*_1_ = 3.95 ns and *τ*_2_ = 49.80 ns for the 8 vol% ACN film. The average fluorescence lifetimes (*τ*_ave_) of the films were subsequently derived according to the following equation:

(2) $$\tau_{ave}=\frac{A_{1}\tau_{1}^{2}+A_{2}\tau_{2}^{2}}{A_{1}\tau_{1}+A_{2}\tau_{2}}$$

The average fluorescence lifetimes of the CsPbBr_3_ films with 0 vol%, 2 vol%, 5 vol%, and 8 vol% of ACN were determined to be 19.76 ns, 23.47 ns, 55.01 ns, and 20.91 ns, respectively. The prolonged carrier lifetime observed in PL/TRPL measurements indicates suppressed non-radiative recombination in the 5 vol–ACN film, providing indirect evidence for mitigated trap-assisted recombination losses. It should be noted that carrier lifetime is also governed by morphological characteristics, interfacial properties, and carrier-density-dependent effects. Consequently, quantitative trap-density characterization techniques, such as space-charge limited current (SCLC) measurements, are indispensable for the direct assessment of trap states. Ultraviolet photoelectron spectroscopy (UPS) measurements were conducted to further elucidate the photogenerated electron transport properties of the CsPbBr_3_ films. Figure 5d illustrates the onset energy (*E_onset_*) region and the cutoff energy (*E_cutoff_*) region of the films with 0 vol% and 5 vol% ACN. Upon ACN incorporation, both *E_onset_* and *E_cutoff_* remained unchanged at 1.86 eV and 16.45 eV, respectively, indicating that the energy band positions were unaffected by the additive. The Fermi level (*E_F_*) was calculated to be −4.77 eV by subtracting the helium I (He I) excitation energy (21.22 eV) from the cutoff energy (*E_cutoff_*). As the onset energy (*E_cutoff_*) was 1.86 eV, the VBM values were calculated to be −6.63 eV by subtracting *E_cutoff_* from the *E_F_* [29,30,31]. Analysis of the band gap diagram yielded a band gap (*E*_g_) of 2.33 eV and a conduction band edge (*E*_c_) of −4.30 eV.

**Figure 6:**
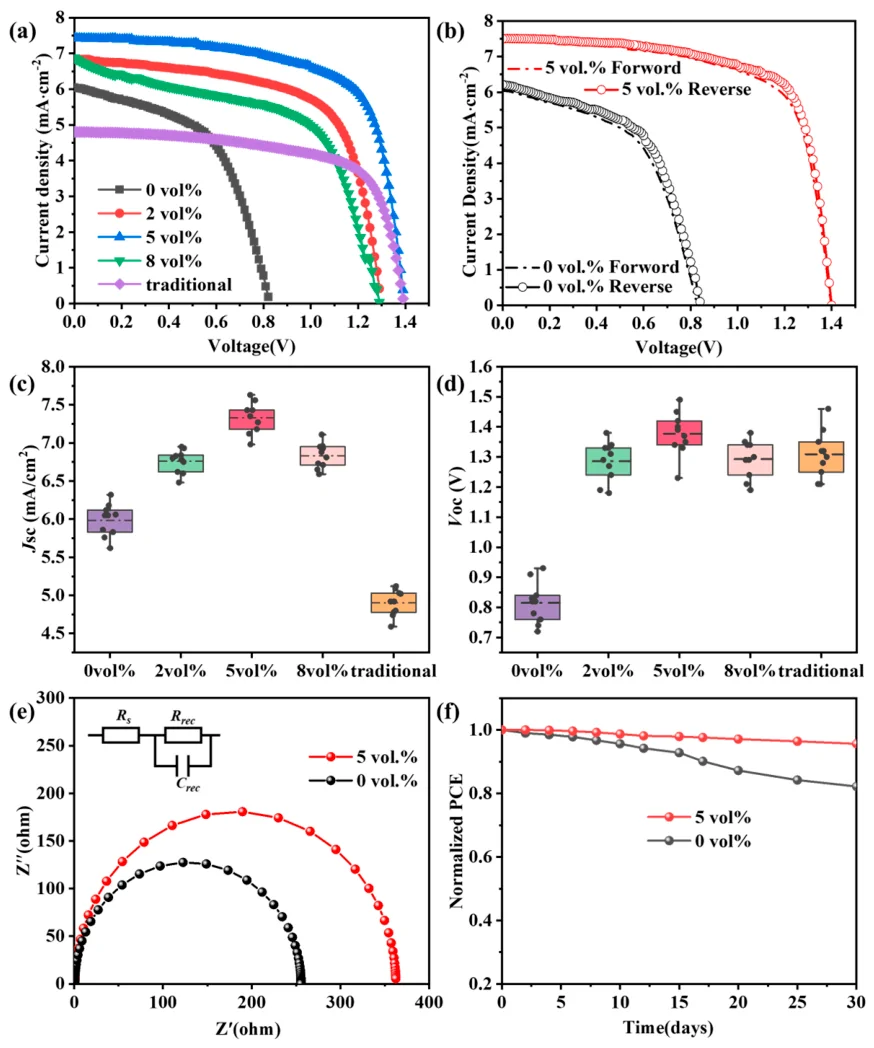
(**a**) *J*-*V* curves of CsPbBr_3_ solar cells fabricated with varying ACN volume fractions; (**b**) the forward and reverse scan *J*-*V* curves; (**c**) box plots of *J*_SC;_ (**d**) box plots of *V*_OC;_ (**e**) Nyquist plots measured under dark conditions, with the equivalent circuit model shown in the inset; and (**f**) environmental stability of devices.

The influence of ACN volume fraction on the photovoltaic performance of CsPbBr_3_ solar cells was systematically investigated. As shown in Figure 6a, the *J*-*V* characteristics of devices fabricated with varying ACN contents were compared against those of a conventionally processed device. The corresponding box plots of *J*_SC_ and *V*_OC_ are presented in Figure 6c,d, respectively. The reference device prepared via the conventional antisolvent method exhibited a PCE of 3.69%, with a *J*_SC_ of 5.80 mA·cm^−2^, a *V*_OC_ of 1.19 V, and an FF of 53.34%. In contrast, direct incorporation of ACN into the precursor solution led to a progressive increase in device efficiency as ACN content increased. Specifically, the device without ACN addition (0 vol%) yielded a PCE of 2.63%, a *J*_SC_ of 6.05 mA·cm^−2^, a *V*_OC_ of 0.82 V, and an FF of 53.91%, whereas the device with 5 vol% ACN achieved significantly improved metrics of 6.69% PCE, 7.35 mA·cm^−2^ *J*_SC_, 1.41 V *V*_OC_, and 64.55% FF. Furthermore, as shown in Figure 6b, the forward and reverse scan curves revealed that the device with ACN exhibited negligible hysteresis, with a hysteresis index (HI) of only 4.78%, whereas the pristine device displayed a more pronounced HI of 7.48%. The performance enhancement at moderate ACN concentrations can be attributed to the formation of high-quality perovskite films characterized by reduced pinhole density and enlarged CsPbBr_3_ grain sizes. This microstructural evolution effectively suppressed non-radiative recombination and prolonged carrier lifetime, thereby boosting overall device performance [32]. However, excessive ACN incorporation altered the solute composition in the precursor solution, inducing heterophase formation during crystallization. This phenomenon impeded CsPbBr_3_ grain growth and introduced pronounced film defects, ultimately compromising device efficiency.

Figure 6e presents the EIS measurements of perovskite films prepared with 0 vol% and 5 vol% ACN, acquired under dark conditions at a bias voltage of 1.1 V, along with the corresponding equivalent circuit diagram. The circuit comprises series resistance (*R_s_*) and interfacial recombination resistance (*R_rec_*) between the perovskite layer and the electrodes. The low-frequency region of the dark-state EIS spectrum exhibits a semicircle corresponding to *R_rec_*, where an increased radius signifies a lower recombination probability. Comparative analysis reveals that the ACN-modified film has a markedly larger semicircle radius. The *R_rec_* increases from 25,511 Ω to 36,170 Ω upon ACN introduction, demonstrating effective suppression of charge carrier recombination. This enhancement is expected to improve the photovoltaic device’s performance [33,34,35]. To investigate the effect of ACN incorporation on the device stability, two sets of unpackaged CsPbBr_3_ PSCs were subjected to stability tests for 30 days under ambient conditions. As shown in Figure 6f, the ACN-modified device retains 95.6% of its initial efficiency after 30 days, whereas the pristine device degraded to 82.2% of its initial efficiency. These results indicate that ACN modification significantly enhances the long-term stability of the device.

## 4. Conclusions

A series of inorganic CsPbBr_3_ films was fabricated by directly incorporating ACN into a PbBr_2_ precursor solution at controlled volume fractions. Comprehensive characterization of the as-prepared ACN-doped CsPbBr_3_ films, including phase structure, microstructure, elemental composition, optical properties, bandgap, and valence band position, revealed that the introduction of an optimal ACN concentration promoted grain growth, diminished grain boundary density and defect states, enhanced light-harvesting capability, and prolonged the average carrier fluorescence lifetime. The champion CsPbBr_3_ solar cell employing a 5 vol% ACN additive achieved a PCE of 6.69%, with a *J*_SC_ of 7.35 mA·cm^−2^, a *V*_OC_ of 1.41 V, and an FF of 64.55%.

## Figures and Tables

**Table 1:** Summarized XRD fitting parameters for pristine and ACN-modified samples, including diffraction angle, FWHM with fitting uncertainty, and Scherrer-calculated coherent scattering crystallite domain size.

	hkl	2θ (°)	FWHM (°2θ)	FWHM Uncertainty	D (nm)	D Error (nm)
Pristine	(110)	21.498	0.119	0.004	385	20
	(200)	30.698	0.108	0.006		
	(211)	37.603	0.309	0.004		
5 vol% ACN	(110)	21.498	0.119	0.004	440	18
	(200)	30.698	0.089	0.005		
	(211)	37.603	0.293	0.004		

**Table 2:** Grain size statistics for the CsPbBr_3_ films fabricated with varying ACN volume fractions.

ACN (vol%)	Grains Number	Minimum (nm)	Maximum (nm)	Average Size (nm)
0	82	150	680	410
2	87	180	760	440
5	104	200	950	520
8	93	160	730	410

**Table 3:** XPS fitting parameters for CsPbBr_3_ films with and without ACN treatment.

	Peak Assignment	Binding Energy (eV)	FWHM (eV)	χ^2^
Pristine	Cs 3d_5_/_2_ (Cs^+^)	724.2	2.25	1.11
	Cs 3d_3_/_2_ (Cs^+^)	738.1	2.26	1.11
	Pb 4f_7_/_2_ (Pb^2+^)	138.4	1.58	1.09
	Pb 4f_5_/_2_ (Pb^2+^)	143.3	1.54	1.09
	Br 3d_5_/_2_ (Br^−^)	68.2	1.82	1.16
	Br 3d_3_/_2_ (Br^−^)	69.2	1.82	1.16
5 vol% ACN	Cs 3d_5_/_2_ (Cs^+^)	724.1	1.89	1.06
	Cs 3d_3_/_2_ (Cs^+^)	738.0	1.90	1.06
	Pb 4f_7_/_2_ (Pb^2+^)	138.3	1.52	1.03
	Pb 4f_5_/_2_ (Pb^2+^)	143.2	1.52	1.03
	Br 3d_5_/_2_ (Br^−^)	68.1	1.60	1.07
	Br 3d_3_/_2_ (Br^−^)	69.1	1.60	1.07

## Data Availability

The original contributions presented in this study are included in the article. Further inquiries can be directed to the corresponding author.
